# Mung Bean Nuclease Treatment Increases Capture Specificity of Microdroplet-PCR Based Targeted DNA Enrichment

**DOI:** 10.1371/journal.pone.0103491

**Published:** 2014-07-24

**Authors:** Zhenming Yu, Kajia Cao, Tanya Tischler, Catherine A. Stolle, Avni B. Santani

**Affiliations:** 1 Division of Genomic Diagnostics and Department of Pathology and Laboratory Medicine, Children's Hospital of Philadelphia, Philadelphia, Pennsylvania, United States of America; 2 Department of Pathology and Laboratory Medicine, Perelman School of Medicine, University of Pennsylvania, Philadelphia, Pennsylvania, United States of America; Deutsches Krebsforschungszentrum, Germany

## Abstract

Targeted DNA enrichment coupled with next generation sequencing has been increasingly used for interrogation of select sub-genomic regions at high depth of coverage in a cost effective manner. Specificity measured by on-target efficiency is a key performance metric for target enrichment. Non-specific capture leads to off-target reads, resulting in waste of sequencing throughput on irrelevant regions. Microdroplet-PCR allows simultaneous amplification of up to thousands of regions in the genome and is among the most commonly used strategies for target enrichment. Here we show that carryover of single-stranded template genomic DNA from microdroplet-PCR constitutes a major contributing factor for off-target reads in the resultant libraries. Moreover, treatment of microdroplet-PCR enrichment products with a nuclease specific to single-stranded DNA alleviates off-target load and improves enrichment specificity. We propose that nuclease treatment of enrichment products should be incorporated in the workflow of targeted sequencing using microdroplet-PCR for target capture. These findings may have a broad impact on other PCR based applications for which removal of template DNA is beneficial.

## Introduction

Since the launch of the first commercial massively parallel pyrosequencing platform in 2005, next-generation sequencing technology has transformed genomic medicine in both basic and clinical research fronts [1]. The past few years have seen wide applications of whole exome sequencing and whole genome sequencing in disease gene discovery, clinical molecular diagnostics and personalized medicine [1]–[7]. However, despite the decreasing cost of next generation sequencing, whole exome sequencing and whole genome sequencing remain expensive especially when high depth of coverage is needed. In addition, significant portions of the exome or genome are not sufficiently sequenced and coverage gaps make the overall variant detection sensitivity of these technologies less than optimal at the current stage [8], [9]. Targeted DNA enrichment coupled with next generation sequencing allows interrogation of relevant genomic regions at high depth of coverage in a cost-effective manner and is well suited for applications such as molecular diagnosis of diseases with complex but defined genetic etiologies [10].

Several approaches are now available for enriching select regions in the genome for sequencing, each with its unique advantages and disadvantages [11]–[14]. Among these, microdroplet polymerase chain reaction (PCR) allows simultaneous amplification of up to thousands of target regions through highly multiplexed microfluidic PCR in picoliter reaction volumes [15]. This straightforward enrichment approach usually yields deep and even coverage and is particularly well suited for capturing small target regions [12], [13], [16]. Amplified PCR products from microdroplet-PCR enrichment are usually end-repaired, concatenated through ligation and then processed into platform-specific libraries for sequencing. This method has been widely adopted for both research and clinical applications by many groups including our own [17]–[24].

On-target efficiency measures how specific a capture method is in enriching target regions in the context of the whole genome [13], [18], [25]. It is always desirable to improve on-target efficiency for any capture method as it is directly related to how much data throughput is needed to achieve a certain depth of on-target coverage [13], [18], [25]. This becomes even more critical in cases such as detection of rare somatic variants in the oncology setting where ultra-deep on-target coverage is needed [26]–[29].

Here we show that carryover of single-stranded template genomic DNA from microdroplet-PCR contributes significantly to off-target reads in resultant libraries. More importantly, treatment of enriched DNA with the mung bean nuclease, an endonuclease specific to single-stranded DNA or RNA [30], can dramatically reduce genomic DNA carryover and increase on-target efficiency of the resultant library. We propose that nuclease treatment of enrichment products should be incorporated in the workflow of targeted sequencing using microdroplet-PCR for enrichment. Our findings may have broad impact on other PCR based applications for which removal of template DNA is beneficial.

## Material And Methods

### Ethics Statement

De-identified patient DNA samples left over from previous genetic tests were used for evaluating performance metrics for targeted DNA enrichment. Since the research did not involve interaction with either subjects or their private identifiable information, the use of the samples did not meet the definition of human subjects research as defined in 45 CFR 46.102(f) and therefore, IRB review and informed consent were not required according to the IRB policies of the Children's Hospital of Philadelphia.

### Target enrichment using the RainDance microdroplet-PCR technology

Target regions of this study include all genomic regions covered by PCR amplicons spanning the coding exons of 11 genes involved in pathogenesis of Noonan spectrum disorders. Primers used to amplify these genes were designed using the RainDance primer design pipeline (Table S1).

Target regions were captured using the RainDance microdroplet-PCR technology following RainDance's protocol. Briefly, 3 µg of human genomic DNA was sheared into 2–5 kb fragments using the Covaris S2 (Covaris). The PCR master mix was made by combining 1.5 µg of the above sheared DNA fragments, 4.7 µl of 10× High-Fidelity Buffer (Life Technologies), 1.26 µl of MgSO4 (Life Technologies), 1.6 µl of 10 mM dNTP (New England Biolabs), 3.6 µl of 4 M Betaine (Sigma), 3.6 µl of RDT Droplet Stabilizer (RainDance Technologies), 1.8 µl of DMSO (Sigma), 0.7 µl 5 units/µl of Platinum High-Fidelity Taq (Life Technologies), and nuclease-free water to bring to a final reaction volume of 25 µl. The master mix was partitioned into microdroplets and merged on-chip with microdroplets of custom primer libraries (RainDance Technologies) using the RainDance 1000 (RainDance Technologies). The emulsion containing merged microdroplet for each sample was dispensed into separate PCR tubes and thermal cycled using the following profile: 94°C for 2 min, 55 cycles at 94°C for 15 s, 54°C for 15 s, 68°C for 30 s, final extension at 68°C for 10 min, and 4°C hold. After PCR amplification, the emulsion was broken by adding 50 µl of RDT 1000 Droplet destabilizer (RainDance Technologies), vortexing for 15 s and spinning at 13,000×g for 5 min. The bottom oil phase was carefully removed from the sample and the remaining sample was purified using a MinElute column (Qiagen) following Qiagen's MinElute PCR Purification protocol. The sample was eluted in 12 µl of EB buffer. 1 µl of the elute was analyzed on a DNA 1000 chip (Agilent) using the Bioanalyzer 2100 (Agilent) to verify consistency of the histogram pattern and quantify the concentration of the enrichment products.

### Nuclease treatment of enriched RainDance PCR products

100 ng of RainDance PCR products were mixed with 2.5 µl of 10× mung-bean nuclease buffer (New England Biolabs), 2 µl of 10 units/µl mung-bean nuclease (New England Biolabs) and nuclease-free water to bring to a final reaction volume of 25 µl. The digestion reaction was incubated at 30°C for 30 min. After nuclease treatment, DNA was purified using 37.5 µl of Agencourt Ampure XP beads (Beckman Coulter) following manufacturers' instructions and eluted in 42.5 µl of nuclease-free water. Untreated samples consisting of 100ng aliquots of captured DNA from the same batch of RainDance enrichment served as paired controls.

### Concatenation of enriched DNA, TruSeq library prep and sequencing using the Illumina Miseq

For end repair, 100 ng of enrichment products, either treated or untreated with mung bean nuclease, were mixed with 10 µl of NEBNext end repair buffer (New England Biolabs), 5 µl of NEBNext end repair enzyme mix (New England Biolabs) and nuclease-free water to bring to a final reaction volume of 100 µl. End repair reaction was incubated at 37°C for 20 min. End-repaired DNA was purified using 150 µl of Agencourt Ampure XP beads (Beckman Coulter) following manufacturer's instructions and eluted in 37.5 µl of nuclease-free water. For concatenation by ligation, 35 µl of end repaired DNA was mixed with 10 µl of 5× NEBNext Quick ligation buffer (New England Biolabs) and 5 µl of T4 DNA ligase (New England Biolabs). Ligation reaction was incubated at 20°C for 20 min. Concatenated DNA was purified using 75 µl of Agencourt Ampure XP beads (Beckman Coulter) following manufacturer's instructions and eluted in 52.5 µl of nuclease-free water.

50 µl of above concatenated DNA was used as the starting material for Illumina TruSeq sequencing library preparation using the TruSeq DNA LT Sample Prep Kit (Illumina) following the Illumina's TruSeq DNA Sample Preparation Guide. Resultant TruSeq libraries were quantified using the Qubit dsDNA BR kit (Life Technologies) and the dsDNA D1K TapeStation kit (Agilent) following manufacturers' instructions. TruSeq libraries were sequenced on MiSeq following Illumina's MiSeq System User Guide. Up to 16 differentially indexed libraries were pooled in equal molar ratio, denatured and diluted to 9 pM and then sequenced on the MiSeq (Illumina) using the 2×150 bp paired-end sequencing kit (Illumina). Nuclease-treated samples and their respective untreated paired controls were processed and sequenced in parallel to rule out batch-specific effects.

### Data analysis using the NextGENe software

FASTQ data generated on the MiSeq was analyzed using the NextGENe software (SoftGenetics). Briefly, FASTQ data was first converted to FASTA data and aligned to the reference human genome hg19. A bed file containing coordinates of the target regions (Table S2) was then applied to analyze on-target efficiency and generate coverage statistics. To detect variants potentially relevant to the pathogenesis of Noonan spectrum disorders from the next generation sequencing dataset, mutation reports were generated for variants within exonic and splicing regions of the 11 Noonan related genes (Table S3). Parameters used in the NextGENe software for data analysis are available upon request.

## Results

### On-target efficiency for libraries derived from DNA enriched through RainDance microdroplet-PCR

Here we define on-target efficiency as the fraction of total number of reads mapped to the target regions compared to the total number of reads mapped anywhere in the genome. We analyzed on-target efficiency for 3 samples enriched through RainDance microdroplet-PCR. As shown in table 1, on-target efficiency ranged from 15.9% to 34.2% with an average of 27.5%. These data suggest that a significant portion of sequencing reads align to regions in the genome other than the intended target regions and therefore represent a waste of sequencing throughput.

**Table 1:** On-target efficiency for libraries prepared from untreated RainDance captured DNA.

Sample ID	Total reads	Aligned reads	Reads on target	On-target efficiency
**1**	2193674	1888037	609182	32.3%
**2**	2002723	1747191	598175	34.2%
**3**	4516825	4245272	674674	15.9%

### Single-stranded genomic DNA carryover is converted to double stranded DNA through DNA synthesis during end repair

**Figure 1:**
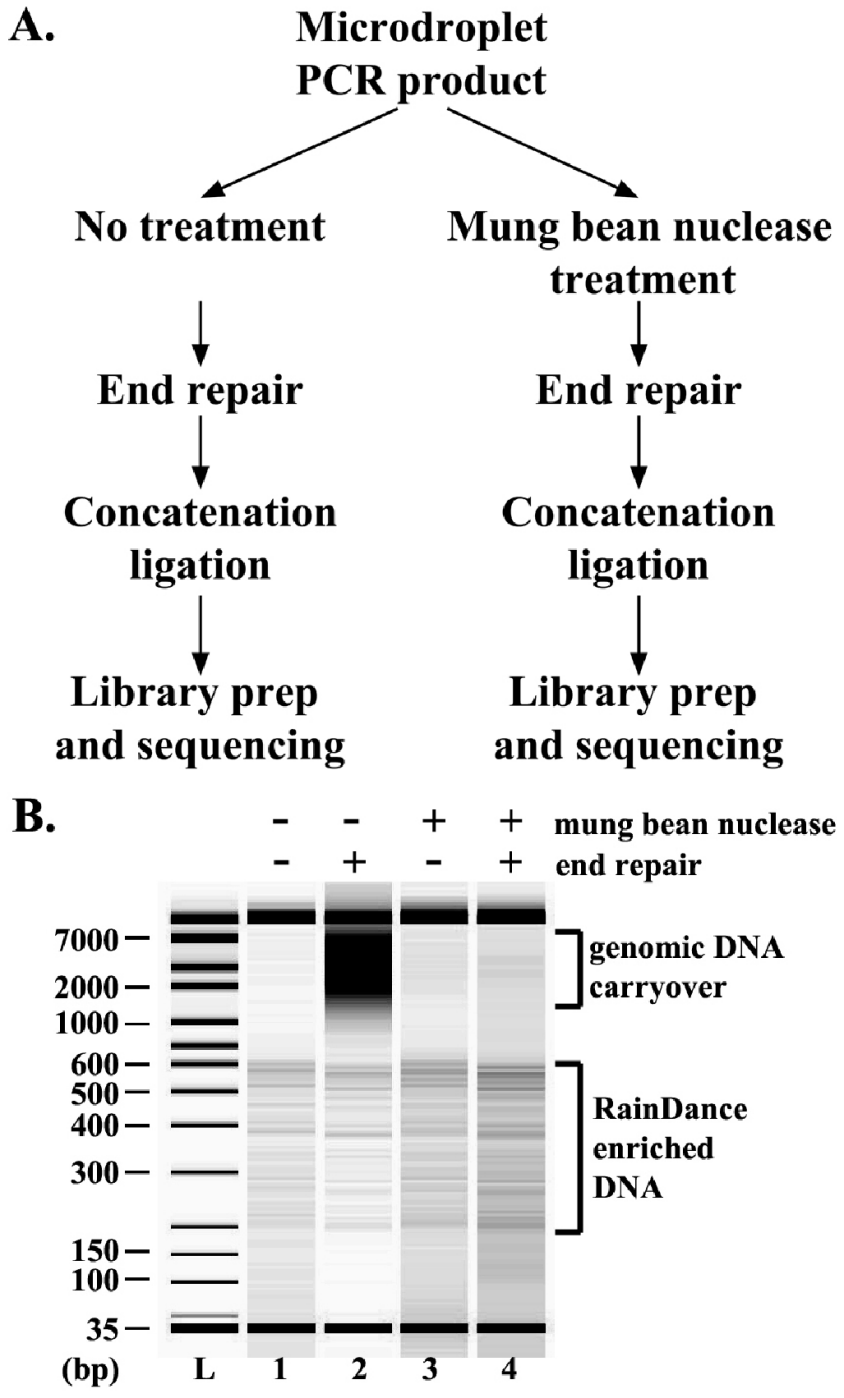
Mung bean nuclease treatment abolished the high molecular DNA smear in RainDance captured DNA after end repair. **A.** An overview of the workflow. Aliquots of 100ng of DNA enriched through microdroplet-PCR were either treated with mung-bean nuclease or untreated as a control. The differentially treated aliquots were end-repaired, concatenated, processed into TruSeq libraries and sequenced on MiSeq in parallel to rule out any batch effect. **B.** Electrophoresis analysis of DNA samples on a high-sensitivity DNA chip using the Agilent 2100 Bioanalyzer. DNA samples in all 4 lanes were derived from 200pg of the same batch of captured DNA. From left to right, the lanes are, “L” lane-the DNA size ladder, lane 1-untreated DNA enriched by RainDance microdroplet-PCR prior to end repair, lane 2- untreated DNA enriched by RainDance microdroplet-PCR post end repair, lane 3- mung bean nuclease treated DNA enriched by RainDance microdroplet-PCR prior to end repair, lane 4- mung bean nuclease treated DNA enriched by RainDance microdroplet-PCR post end repair.

Carryover of template genomic DNA has been previously reported to cause off-target reads and reduce on-target efficiency [15], [24]. We thus examined whether genomic DNA contamination is present in our enriched DNA samples and subsequently leads to the low on-target efficiency observed in Table 1. Since template genomic DNA fragments are 2–5kb in size while enrichment amplicons primarily range from 128bp-600bp in size (Table S1), we expect that these two populations of DNA molecules should be readily distinguishable through electrophoresis on a Bioanalyzer high sensitivity DNA chip. However, we did not detect significant genomic DNA carryover with electrophoresis analysis (lane 1 in Figure 1B). At first glance, this result seems to contradict our initial hypothesis that genomic DNA carryover is present in the enrichment product. However, we considered the possibility that template genomic DNA may have remained in the denatured and primarily single-stranded state even after microdroplet-PCR. Since the dye in the Bioanalyzer dsDNA kit only specifically binds double-stranded DNA, single-stranded DNA molecules are thus “invisible” on the chip.

Indeed, we observed a high molecular weight smear resembling sheared template genomic DNA in captured DNA after it had gone through end-repair (Figure 1A and Figure 1B, lane 2). This is consistent with the possibility that single-stranded template genomic DNA molecules are converted into double-stranded DNA during the end-repair reaction by DNA polymerase. To further examine the hypothesis that the high molecular weight smear originated from single-stranded DNA, we treated captured DNA prior to the end repair step with mung bean nuclease, an endonuclease specific for single-stranded DNA or RNA [30], [31]. As shown in Figure 1B (lane 4), nuclease treatment abolished majority of the high molecular weight smear in the enriched DNA after end repair. In contrast, enriched DNA was spared from mung bean nuclease digestion, suggesting that it remained double-stranded and thus resistant to mung bean nuclease digestion (Figure 1, lane 3 and 4). Taken together, these results suggest that at least part of genomic template DNA carryover in captured DNA exists as single-stranded DNA and is converted to double-stranded DNA during the end-repair reaction.

### Treating captured DNA with the mung bean nuclease increases on-target efficiency

**Figure 2:**
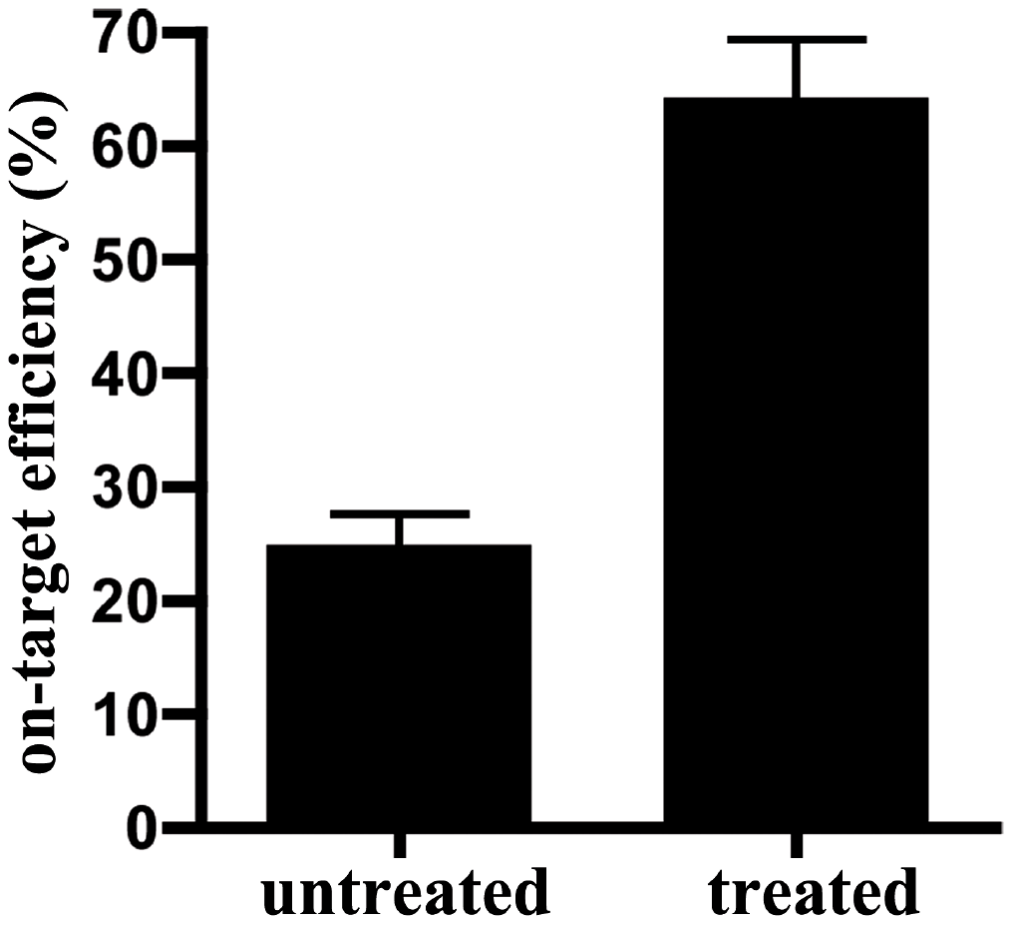
Mung bean nuclease treatment significantly increases on-target efficiency for DNA enriched through RainDance microdroplet-PCR. Aliquots of RainDance enriched DNA for the same sample were either treated or not treated with mung bean nuclease, processed into TruSeq libraries, and sequenced on MiSeq as illustrated in Figure 1A. Plotted is the mean value of on-target efficiency of 3 samples that went through parallel treatments (also see Table 2). Nuclease treatment leads to significantly higher on-target efficiency (*p = 0.018, one-tail paired *t* test; error bar, SEM).

If single-stranded genomic DNA carryover contributes to off-target reads in the resultant library, mung bean nuclease treatment, which selectively digests and removes single stranded DNA, should alleviate off-target reads and improve on-target efficiency. To test this hypothesis, we sequenced libraries prepared from the same sample of enriched DNA, either with or without mung bean nuclease treatment prior to end repair (workflow illustrated in Figure 1A). As shown in Table 2, upon mung bean nuclease treatment, on-target efficiency in resultant libraries increased 2.1- to 3.8-fold among the 3 samples analyzed, which is statistically significant (Figure 2). These data further prove that at least part of off-target reads can be attributed to single-stranded template genomic DNA carryover from microdroplet-PCR. In addition, these results demonstrate that treatment of microdroplet-PCR enriched DNA with a nuclease specific to single stranded DNA, such as mung bean nuclease, is a highly effective way to diminish off-target reads and improve on-target efficiency in the resultant library.

**Table 2:** On-target efficiency for libraries prepared from samples either treated or untreated with the mung bean nuclease.

Sample ID	Nuclease treatment	Total reads	Aligned reads	Reads on target	On-target efficiency	Fold increase
**4**	No	2040068	1888854	513149	27.2%	2.19
	Yes	2176298	1798070	1069568	59.5%	
**5**	No	2344430	2158492	603053	27.9%	2.09
	Yes	1847463	1499265	877131	58.5%	
**6**	No	4185191	3917002	759764	19.4%	3.84
	Yes	3360424	2907096	2164377	74.5%	

### Mung bean nuclease treatment has no negative impact on variant detection

To address the utility of nuclease treatment of captured DNA in applications such as detection of variants for clinical diagnostics, we compared mutation reports generated using the NextGENe software for the same sample either treated or not treated with mung bean nuclease. As shown in Table 3, all variants detected with the traditional protocol were also detected with the modified protocol that incorporated nuclease treatment. Moreover, as a result of increased on-target efficiency and thereby coverage, additional variants were detected in samples 4 and 5 when treated with mung bean nuclease. Both of additional variants were later confirmed by Sanger sequencing (data not shown). These results suggest that mung bean nuclease treatment has no apparent negative impact on variant detection and instead improves detection of variants in the resultant library prep.

**Table 3:** Comparison of variants detected for the same sample either treated or not treated with mung bean nuclease.

Sample ID	Nuclease Treatment	Index	Chr. Position	Gene	Coverage	Score	Mutation Call	Amino Acid Change
**4**	Not treated	1	2∶39294787	SOS1	983	23.9	T>GT	65R>RR
		2	7∶140449150	BRAF	4274	28.1	T>CT	643G>GG
		3	11∶534242	HRAS	1668	23.9	A>AG	27H>HH
		4	11∶119145667	CBL	2011	23.4	A>AG	
		5	11∶119170362	CBL	1395	24	C>CT	864L>LL
		6	12∶25362777	KRAS	1247	22.9	A>AG	
		7	19∶4110552	MAP2K2	130	14	C>CG	135G>GG
		8	19∶4117528	MAP2K2	1219	22.4	G>AG	64V>VV
	Treated	1	2∶39294787	SOS1	2322	24.4	T>GT	65R>RR
		2	7∶140449150	BRAF	7266	30	T>CT	643G>GG
		3	11∶534242	HRAS	2615	27.2	A>AG	27H>HH
		4	11∶119145667	CBL	5181	26	A>AG	
		5	11∶119170362	CBL	2569	27.2	C>CT	864L>LL
		6	12∶25362777	KRAS	4268	28.9	A>AG	
		7*	12∶25368462	KRAS	4	1.2	C>T	161R>R
		8	19∶4110552	MAP2K2	258	17.4	C>CG	135G>GG
		9	19∶4117528	MAP2K2	2531	26.6	G>AG	64V>VV
**5**	Not treated	1	2∶39249915	SOS1	938	23.3	T>CT	552R>GR
		2	2∶39294787	SOS1	1163	24.5	T>GT	65R>RR
		3	11∶534242	HRAS	1788	25.5	A>G	27H>H
		4	12∶25362777	KRAS	1560	25.4	A>AG	
		5	19∶4101062	MAP2K2	3810	28.3	G>GT	220I>II
	Treated	1	2∶39249915	SOS1	2607	24.4	T>CT	552R>GR
		2	2∶39294787	SOS1	2253	24.9	T>GT	65R>RR
		3	11∶534242	HRAS	1240	24	A>G	27H>H
		4	12∶25362777	KRAS	3890	28.6	A>AG	
		5*	12∶25368462	KRAS	8	7.2	C>T	161R>R
		6	19∶4101062	MAP2K2	2139	25.2	G>GT	220I>II
**6**	Not treated	1	11∶534242	HRAS	294	19.6	A>AG	27H>HH
		2	12∶25368462	KRAS	21	10.3	C>T	161R>R
		3	15∶66782048	MAP2K1	1502	25.3	C>CT	
	Treated	1	11∶534242	HRAS	807	20.6	A>AG	27H>HH
		2	12∶25368462	KRAS	67	14.3	C>T	161R>R
		3	15∶66782048	MAP2K1	5411	27.7	C>CT	

*: Variants detected only when samples were treated with mung bean nuclease.

## Discussion

Microdroplet-PCR-based enrichment is among the most commonly used capture methods for targeted next generation sequencing [12]. On-target efficiency is an important performance metric for measuring specificity of the target enrichment strategy. Lower on-target efficiency means that more sequencing throughput and thus higher associated cost are required to achieve the same depth of on-target coverage. Although some studies found that microdroplet-PCR can achieve high capture specificity [15], experience from other studies including our own suggest there is still much room to improve upon on-target efficiency for this enrichment method [18], [21]–[24], [32]. We found that carryover of single-stranded genomic DNA is a major contributing factor for off-target reads in the targeted libraries enriched using the microdroplet-PCR technology. Moreover, treatment of captured DNA with single-stranded DNA specific endonucleases such as the mung bean nuclease, effectively removes genomic DNA carryover and thereby improves on-target efficiency without affecting integrity of variant detection (Tables 2 and 3).

Genomic DNA carryover has been known to adversely affect on-target efficiency since the early development stage of the microdroplet PCR technologies [15]. Here we demonstrated that at least part of genomic DNA carryover from RainDance microdroplet enrichment exists in the single-stranded state. Given the complexity of the template genomic DNA and its relatively high concentration in the final enriched product, it is conceivable that the single-stranded genomic DNA molecules may interact with each other to form short stretches of double-stranded DNA through low-stringency base pairing at relatively low temperatures. This may provide the structural basis for DNA polymerase mediated DNA synthesis through primer extension during end repair, resulting in the high molecular smear observed in the post end repair reaction (Figure 1). Once converted into double-stranded DNA, template genomic DNA carryover is processed together with captured DNA into the final sequencing library, leading to off-target reads.

In further support of our hypothesis, treatment of DNA enriched through microdroplet-PCR with mung bean nuclease, an endonuclease specific for single stranded DNA [30], [31], diminished the high molecular weight smear observed in the post end-repair enrichment DNA. Nuclease treatment improved on-target efficiency in the resultant library. However, a significant amount of off-target reads still exist even if the enriched DNA has been treated with mung bean nuclease (Table 2). It is possible that a low level of genomic DNA carryover may anneal and form double stranded DNA which leads to off target reads. In addition, potential non-specific amplification during microdroplet-PCR may contribute to off target reads. Consistent with some previous observations [24], [32], on-target efficiency varies significantly among samples enriched through microdroplet PCR in this study (Tables 1 and 2). This may at least partially be attributed to versatility of relative amount of genomic DNA carryover in various enriched samples.

Mung bean nuclease digestion was previously used to remove PCR primers for direct sequencing of double-stranded PCR products without fragment purification [33]. We show here that template genomic DNA carryover in microdroplet-PCR products exists as denatured single-stranded DNA and therefore can also be removed by mung bean nuclease digestion. Treatment of PCR products with mung bean nuclease may improve on-target efficiency for other PCR based enrichment methods, such as Fluidigm Access Array, when a similar post-enrichment protocol is used [21], [34]. One way to circumvent off-target reads derived from single-stranded template genomic DNA is to fuse sequencing platform-specific adapter sequences to locus-specific PCR primers and thereby generate amplicon libraries directly from these primers through PCR [26], [28], [35]. However, there are limitations associated with such enrichment strategies including the need for adapter sequences and sequencing platforms to be preconfigured and fixed. In addition, the size of the amplicon library is limited by read length of the sequencing kit [34].

To alleviate genomic DNA carryover in microdroplet-PCR enriched DNA, Tewhey *et al* biotinylated genomic DNA through nick translation and subsequently removed it from enrichment product using streptavidin-coated beads [15]. Sivakumaran *et al* had also tried gel fractionation to purify RainDance enriched PCR products prior to end repair [24]. In comparison, nuclease treatment is a straightforward way to remove genomic DNA carryover and is fully compatible with automation of library preparation. We propose that nuclease treatment of DNA enriched through microdroplet-PCR should be incorporated into the workflow for sequencing library preparation to improve on-target efficiency.

## Supporting Information

Table S1 **Primer sequences for microdroplet PCR and the length of resultant amplicons.**
(XLSX)

Table S2 **Hg19 genomic coordinates of regions of interest for calculation of on-target efficiency.**
(XLSX)

Table S3 **Hg19 genomic coordinates of exons with flanking splicing sites of genes associated with Noonan spectrum disorders.**
(XLSX)
